# Dr. S. P. Hullihen

**Published:** 1857-05

**Authors:** 


Died, at Wheeling, Va., March 27th, 1857, S. P. Hullihen, M.D., aged forty-six years. Dr. Hullihen was a graduate of Washington Medical College, of Baltimore, and early in professional life devoted himself to the specialty of Surgery, in which he reached great distinction. He chiefly confined his practice to minor surgery, including the operations of the dentist, the oculist, and general surgery of the head. In these his fame and popularity had extended far beyond Wheeling, and his death is felt to be a public calamity in the whole section of country where his usefulness has made him beloved.

The death of Dr. Hullihen will be a great loss, especially to the profession of the dentist, in which he was eminent, and in which he had made important discoveries and inventions. But justice cannot be done now to his fame or his virtues, and we hope to furnish a more worthy and extended notice of him in a future number.

